# Role of Sentinel Lymph Node Biopsy for Skin Cancer Based on Clinical Studies

**DOI:** 10.3390/cancers15133291

**Published:** 2023-06-22

**Authors:** Shoichiro Ishizuki, Yoshiyuki Nakamura

**Affiliations:** Department of Dermatology, Faculty of Medicine, University of Tsukuba, 1-1-1 Tennodai, Tsukuba 305-8575, Japan

**Keywords:** skin cancer, surgical margin, lymph node dissection, skin graft

## Abstract

**Simple Summary:**

Sentinel lymph nodes are the first regional lymph nodes to receive lymph fluid from the primary cancer, sentinel lymph node biopsy (SLNB) can detect occult nodal metastasis. In addition, it may also have a therapeutic effect via regional disease control. SLNB was first introduced in melanoma among skin cancers and is currently used in many types of skin cancers. Previous randomized clinical trials suggested a prognostic benefit from SLNB in melanoma patients. However, whether SLNB affects the prognosis of patients with nonmelanoma skin cancer remains largely unknown, although SLNB may provide important information about nodal status. Since SLNB may be associated with adverse events including infection, lymphedema, and nerve injury, routine application of SLNB for all patients with skin cancer is not recommended. In this review, we summarize the evidence available in the literature regarding the role of SLNB in skin cancer.

**Abstract:**

The sentinel lymph node is the first lymph node from the primary tumor. Sentinel lymph node biopsy (SLNB) is a surgical procedure that can detect occult nodal metastasis with relatively low morbidity. It may also have a therapeutic effect via regional disease control. The Multicenter Selective Lymphadenectomy-I (MSLT-I) trial revealed a prognostic benefit from SLNB in melanoma patients. However, it remains unclear whether there is a prognostic benefit from SLNB in patients with nonmelanoma skin cancer owing to a lack of randomized prospective studies. Nevertheless, SLNB provides important information about nodal status, which is one of the strongest factors to predict prognosis and may guide additional nodal treatment. Currently, SLNB is widely used in the management of not only patients with melanoma but also those with nonmelanoma skin cancer. However, the utilization and outcomes of SLNB differ among skin cancers. In addition, SLNB is not recommended for routine use in all patients with skin cancer. In this review, we provide a summary of the role of SLNB and of the indications for SLNB in each skin cancer based on previously published articles.

## 1. Introduction

Sentinel lymph nodes (SLNs) are the first regional lymph nodes to receive lymph fluid from the primary cancer. SLNs can be detected by use of planar lymphoscintigraphy, single-photon emission computed tomography/computed tomography (SPECT/CT), and blue dye and indocyanine green fluorescence, and the combined use of these techniques has been demonstrated to improve the accuracy of SLN identification [1,2]. Sentinel lymph node biopsy (SLNB) was first introduced to detect occult LN metastases in melanoma among skin cancers [3,4]. Since previous studies have shown that metastasis of SLNs is a critical predictive factor for worse prognosis in various skin cancers [5,6,7,8,9,10], early detection of occult metastasis in SLNs and subsequent management for potentially metastatic non-SLNs might improve the prognosis. Indeed, a previous randomized trial revealed a beneficial impact of SLNB for the prognosis of melanoma patients [11,12]. Indication of SLNB has also been expanded for other skin cancers, and current guidelines recommend SLNB in some nonmelanoma skin cancers [13,14]. However, whether SLNB affects the prognosis of patients with nonmelanoma skin cancer remains largely unknown owing to a lack of randomized prospective studies, even though SLNB is a powerful tool for improving nodal staging and may guide additional nodal treatment. In this review, we summarize the evidence available in the literature pertaining to the role of SLNs in skin cancer.

## 2. Role of SLNB in SKIN Cancer

### 2.1. Melanoma

#### 2.1.1. Role of SLNB and Management after Detection of Positive SLNs in Melanoma

Melanoma is one of the most aggressive malignant skin tumors with high metastatic potential and its incidence has been increasing [15]. Melanoma can be classified into four main subtypes according to the clinical and histologic findings: lentigo maligna melanoma (LMM), superficial spreading melanoma (SSM), acral lentiginous melanoma (ALM), and nodular melanoma (NM) [16]. The proportions of melanoma subtypes differ according to the ethnic population. Whilst rare in white populations, ALM has a much higher incidence in Asian populations: previous studies demonstrated that in white populations, ALM accounts for 1% to 7% of all melanomas, but in Asian populations, for more than 50% of these [17,18,19]. Until the 1990s, elective lymph node dissection (LND) was recommended for melanoma patients with high-risk metastases. However, LND is associated with severe complications, such as lymphedema and lymph fistula. In 1992, the SLNB was introduced by Morton et al. as a surgical method to identify with a high degree of accuracy clinically occult metastases in the regional lymph nodes of melanoma patients [3,4]. SLNB has been shown to be a safe, low-morbidity procedure for identifying the regional nodal status in melanoma, and elective LND was replaced by SLNB [20]. Since then, multiple retrospective studies have investigated the role of SLNB in melanoma, and the status of SLNs has been reported to be one of the strongest prognostic factors in melanoma [21].

The Multicenter Selective Lymphadenectomy-I (MSLT-I) trial was a phase III trial that compared the outcomes of primary tumor excision with SLNB, followed by immediate LND for positive SLNs, with those of tumor excision followed by nodal observation and nodal surgery delayed until detection of nodal metastases [11]. In this study, the mean 10-year disease-free survival (DFS) rate was significantly improved in the biopsy group when compared with that in the observation group among patients with intermediate-thickness melanomas, defined as 1.20–3.50 mm, and those with thick melanomas, defined as >3.5 mm, although the 10-year melanoma-specific survival rate did not differ significantly among patients with intermediate-thickness melanoma or patients with thick melanomas [11]. The authors also conducted a latent-subgroup analysis to estimate the treatment effect of SLN followed by immediate LND in patients with nodal metastases and showed that such management with SLNB significantly improved the 10-year rate of distant disease-free survival and the 10-year rate of melanoma-specific survival for patients with intermediate-thickness melanomas, suggesting a prognosis advantage conferred by management with SLNB [12]. The false-negative rate among patients with negative SLNB results was 5.2% in this study [12]. The false-negative result is thought to be caused by poor techniques to accurately detect and resect SLNs, inadequate pathologic examination, and biologic issues such as obstruction of the lymphatic basin by metastatic tumors [22]. In the second Multicenter Selective Lymphadenectomy (MSLT-II) trial, the usefulness of immediate LND in melanoma patients with SLN metastases was evaluated [23]. This study compared the outcomes of patients with SLN metastases in terms of those with immediate LND and of those with nodal observation using ultrasonography [23]. The DFS was slightly better in the LND group than in the observation group, and this improved DFS was based on an increased rate of disease control in the regional nodes [23]. However, melanoma-specific survival was comparable between the LND group and the observation group [23]. Among the patients of the observation group, most basins were free of nodal recurrence at 10 years, suggesting that SLNB may work not only as an examination for staging but also as a curative treatment for some melanoma patients [24]. DeCOG-SLT, another multicenter, randomized, phase III trial comparing the survival of melanoma patients with a positive sentinel node who underwent immediate LND with those who did not, demonstrated similar results to those of the MSLT-II trial [25,26]. Recent retrospective studies also demonstrated that the recurrence rate and OS or melanoma-specific survival rates in melanoma patients with positive SLNs who underwent immediate LND were comparable to those in patients without immediate LND [27,28]. Collectively, these studies indicate that only resection of metastatic SLNs, but not additional resection of metastatic non-SLNs, might improve survival, although resection of metastatic non-SLNs may contribute to a lower rate of nodal recurrence. In these clinical trials, most patients did not receive immune checkpoint inhibitors or BRAF/MEK inhibitors as adjuvant therapies. These adjuvant treatments have recently shown improvement in the prognosis of melanoma patients with stage III disease [29,30]. Therefore, such adjuvant therapies without immediate LND would be more selected for treatment of melanoma with positive SLNs in the future. However, caution is warranted. These trials comprised mainly white populations, in which SSM and LMM are the major clinical types of melanoma. In contrast, ALM, the major clinicopathologic type of melanoma in Asian populations, would comprise only a small portion of the participants. ALM has been shown to be less susceptible to immune checkpoint inhibitors, and BRAF mutation is rare in ALM [31,32,33]. Therefore, the role of immediate LND after detection of positive SLNs in ALM remains unclear, and further prospective studies focusing on ALM are necessary.

Immune checkpoint inhibitors and BRAF/MEK inhibitors may cause various adverse events, and fatal cases with the adverse events associated with these treatments have also been reported [34,35]. Because of the possibility of adverse events, choice of adjuvant therapy should be determined with careful consideration of the patient’s prognosis and the treatment’s toxicity. The number of metastatic lymph nodes is known to be an important prognostic factor [36]. However, detection of the exact number of lymph nodes with occult metastasis is difficult unless LND is performed. In contrast, non-SLN metastases were a strong, independent prognostic factor for recurrence. As for predicting factors for metastases in non-SLNs, the MSLT-I trial demonstrated that primary tumor ulceration, percent nodal metastatic area, maximum metastatic dimension, and extracapsular extension were significantly associated with the presence of non-SLN metastases in the univariate analysis, although no single factors were retained in the multivariate analysis [11]. In a previous prospective study, tumor thickness ≥ 2 mm and tumor burden ≥ 2 mm in metastatic SLNs were significant factors predicting non-SLN status in multivariate analyses [37]. Therefore, these factors may be useful to predict the status of non-SLNs in SLN-positive patients and to determine subsequent treatments.

#### 2.1.2. Predictive Factors for SLN Metastases as Indicators for SLNB Application in Melanoma

As mentioned previously, the SLN status is important not only for tumor staging but also for the decision about adjuvant therapies. However, SLNB has some risk of complication including infection, lymphedema, and nerve injury [38]. General anesthesia may be required for SLNB in some cases. Therefore, whether SLNB should be performed may depend on various factors including age, general condition, and primary tumor location. The National Comprehensive Care Network (NCCN) guidelines recommend SLNB to be considered in melanoma patients with a 5% or higher risk of positivity and offered to those with more than 10% risk of positivity. SLN metastases develop in more than 10% of patients with tumor thickness > 1 mm (pT2–pT4), in more than 5% of patients with tumor thickness between 0.8 mm and 1.0 mm (pT1b), and in less than 5% of patients with tumor thickness < 0.8 mm (pT1a–pT1b) [39,40]. However, recent studies demonstrated that the rate of positive SLNB results increases to more than 5% in thin melanoma with some risk factors. Walker et al. evaluated the rate of positive SLNB results in thin melanoma with thickness ≤ 1 mm and reported that thickness and mitotic rate independently predicted SLNB positivity [41]. Huang et al. conducted a meta-analysis evaluating positivity of SLNB results in thin melanoma and found that features that significantly predicted a positive SLNB result included thickness ≥ 0.8 mm (positive rate, 7.0%), presence of ulceration (positive rate, 4.2%), and mitotic index > 0/mm^2^ (positive rate, 7.7%) [42]. In another retrospective study analyzing the outcomes of patients with pathologic T1a (thickness < 0.8 mm, nonulcerated) who underwent SLNB, factors significantly associated with positive SLNB results were age ≤ 42 years (positive rate, 7.5%), head/neck primary tumor location (positive rate, 9.2%), lymphovascular invasion (positive rate, 21.4%), and mitotic index ≥ 2/mm^2^ (positive rate, 8.2%) [43]. These reported factors associated with an increased rate of positive SLNB results may be useful to determine which patients with thin melanoma should undergo SLNB and are summarized in Table 1. The current NCCN guidelines do not recommend SLNB for all T1 lesions but recommend SLNB to be considered in melanoma patients with T1b lesions (thickness < 0.8 mm with ulceration or 0.8–1 mm) and T1a lesions with thickness < 0.8 mm and with other adverse features such as mitotic index ≥ 2/mm^2^, particularly in the setting of young age and lymphovascular invasion [40].

#### 2.1.3. Role of SLNB Depending on Subtypes of Melanoma

The risk of SLN metastases may differ among subtypes of melanoma. In a retrospective study comparing the rate of positive SLNB results among subtypes of melanoma (SSM, LMM, ALM, NM, and others), ALM was independently associated with the highest risk for positive SLNB results, whereas LMM was associated with the lowest risk among the included subtypes [44]. A subgroup analysis demonstrated that ALM was also independently associated with the highest risk for positivity of SLNB results in both stage IB and stage II disease [44]. These results suggest that SLNB should be more encouraged for patients with ALM than for those with the other subtypes, especially in cases of stage IB or stage II disease. Desmoplastic melanoma (DM), characterized by the presence of desmoplastic components in lesions, is an uncommon type of melanoma that accounts for less than 4% of all cutaneous melanoma [45,46,47]. DM shows similar clinical behavior to soft tissue sarcoma and has a higher risk of local and distant recurrence but a lower risk of nodal recurrence than those of conventional melanoma [48]. DM can be divided into 2 subtypes: pure DM containing more than 90% desmoplastic components and mixed DM containing 10–90% desmoplastic components [47,48]. These subtypes have been reported to show distinct clinical behaviors, and mixed DM is associated with a high rate of lymph node metastases [47,48]. A previous systematic review demonstrated that the overall rate of positive SLNB results was 8.5% [49]. However, the rate of positive SLNB results in patients with pure DM (mean, 4.9%) was significantly lower than that in patients with mixed DM (mean, 14.8%) [49]. The rate of positive SLNB results in patients with pure DM is beneath the threshold of the NCCN guideline recommendation for considering SLNB [40]. Therefore, routine SLNB might not be recommended for all pure DM patients even if the tumors are of ≥T1b, and which factors are associated with increased risk of SLN metastases in pure DM patients should be elucidated in further studies to select which patients should undergo SLNB.

#### 2.1.4. Significance of the Time Interval between Diagnostic Excision-Biopsy and the Outcomes of the SLNB in Melanoma

Several studies focused on the time interval between diagnostic excision-biopsy of primary melanoma and the results of the SLNB. Recently, Sharouni et al. analyzed the outcomes of a large cohort of patients using a Dutch population-based cohort of patients with melanoma who underwent SLNB within 100 days of the initial diagnosis and a similarly specified cohort from a large Australian melanoma treatment center [50,51]. They reported no significant association between time from excision-biopsy to SLNB and positivity of SLNB results in either cohort. In contrast, Dutch patients whose SLNB was conducted in the second or third months after diagnosis showed a significantly larger tumor diameter than that of patients who underwent SLNB in the first month, although such a difference in tumor diameter was not found in the Australian cohort. However, no significant impact of time to SLNB on DFS or OS was found in either cohort. These results indicate that prognosis does not differ depending on the interval if SLNB is performed within 100 days of the initial diagnosis. However, since these patients did not receive the recently developed adjuvant therapies, further studies including data of patients who underwent adjuvant therapies are needed to determine the appropriate timing of SLNB in the new era of melanoma treatment. A prospective clinical study with a large number of participants to evaluate the role of SLNB in melanoma in this new era (NCT04759781) is also ongoing, which might clarify this issue.

### 2.2. Squamous Cell Carcinoma (SCC)

In this review, SCC is divided into cutaneous SCC (cSCC), which develops in nonspecial skin sites, and anogenital SCC, including vulvar SCC, penile SCC, and anal SCC, because anogenital SCC may have different characteristics from those of cSCC, such as a high potential of nodal metastases and frequent association with high-risk HPV infection or lichen sclerosis et atrophicus [52,53].

#### 2.2.1. cSCC

##### Role of SLNB in cSCC

cSCC is characterized by a malignant proliferation of keratinocytes [54]. Whilst the clinical course of cSCC is mild in most cases, it may show locally aggressive behavior and metastatic potential [54,55]. Metastases were found in 2–6% of overall cSCC patients [5,56,57,58]. Metastatic cSCC most commonly involves regional lymph nodes, and presence of metastases in lymph nodes is one of the most important prognostic factors in cSCC patients [5,6,7,8]. Therefore, SLNB is an important tool for improving node staging, although whether SLNB improves the prognosis of cSCC patients remains largely unclear because of a lack of evidence from randomized clinical trials. A randomized clinical study for evaluating the role of SLNB in cSCC (NCT04664582) is ongoing, and this trial may show the impact of SLNB in the prognosis of cSCC patients in the future.

##### Predictive Factors for SLN Metastases as Indicators for SLNB Application and Management after Detection of Positive SLNs in cSCC

Currently, SLNB is not recommended in all cSCC patients. Since the NCCN guidelines recommend SLNB to be considered or offered in melanoma patients with a 5–10% or higher risk of positivity, SLNB should be performed only in high-risk cSCC patients with an estimated positivity of SLNB results being more than 5–10%, and therefore, identifying risk factors for node metastases is important. Previous studies evaluated the risk factors associated with positivity of SLNB results, but the findings differed among the studies, presumably because of the heterogeneity of the surgical indication for SLNB. The overall positive rate and false-negative rate in most of the previous studies were 5.4–14.6% and 0–5.7%, respectively [59,60]. Chabrillac et al. reported that factors significantly associated with increased positivity of SLNB results were tumor size and poor tumor differentiation [59]. Another study showed that a tumor diameter ≥ 2 cm, tumor depth > 6 mm, or invasion into the subcutaneous fat, perineural invasion of nerves with a diameter ≥ 1 mm, moderate or poor histologic differentiation, lymphovascular invasion, and immunosuppression were associated with positive SLNB results [60]. Whereas the eighth edition of the American Joint Committee on Cancer (AJCC-8) is typically used for staging of cSCC, the Brigham and Women’s Hospital (BWH) system has been proposed as an alternative T staging system for determining characteristics predictive of nodal metastases. Schmitte et al. compared these two systems and reported that the BWH system provides more delineation of high-risk cSCC for nodal metastases (Table 2 and Table 3) [61]. In the BWH system, tumors with none of the high-risk features (diameter ≥ 2 cm, poor differentiation, perineural invasion, or invasion beyond the subcutaneous tissues) are classified as T1. T2a tumors have one risk factor, T2b tumors have two or three factors, and T3 tumors have all the risk factors for bone invasion. Tejera-Vaquerizo et al. analyzed the rate of positive SLNB results according to the AJCC-8 and the BWH staging criteria using data from a systematic review (Table 2 and Table 3) [62]. In cSCC of the head and neck, the rates of positive SLNB results for patients with T1, T2a, or T2b disease were 0%, 6.2%, and 29.4%, respectively [62]. None of the patients had T3 disease [62]. In the trunk and extremities, the rates of positive SLNB results for patients with T1, T2a, T2b, or T3 disease were 0%, 4.4%, 45%, and 75%, respectively [62]. Taken together, the findings of these studies suggest that SLNB should be considered for at least T2b patients according to the BWH staging system, whereas SLNB may not be required for T1 patients. However, presence of immunosuppression, which is not included in the risk factors in the BWH system, may be also associated with nodal metastases and should be taken into account when determining whether to perform SLNB. As mentioned above, a randomized clinical study for evaluating the role of SLNB in cSCC (NCT04664582) is ongoing, which may also further clarify which patients with cSCC should undergo SLNB. Where metastatic disease is identified in the SLNs, LND is usually performed. However, given radiosensitive tumors, studies comparing the outcomes of LND and adjuvant radiation therapy (RT) for patients with positive SLNs might be required in the future.

#### 2.2.2. Vulvar SCC

##### Role of SLNB in Vulvar SCC

Vulvar SCC is a relatively rare malignancy. Until recently, standard management for patients with vulvar SCC clinically restricted to the vulva with a depth of invasion > 1 cm had been wide local excision and either ipsilateral or bilateral inguinal LND. The Groningen International Study on Sentinel Nodes in Vulvar Cancer (GROINSS-V) I is a prospective international observational study to evaluate the role of SLNB in early-stage vulvar SCC [63,64,65]. In this study, vulvar SCC patients with a tumor < 4 cm in diameter, with a depth of invasion > 1 mm, and without clinical inguinal lymph node metastases were included. The rate of positive SLNB results was as high as 26.2%, and LND was performed only in patients with positive SLNs. The isolated inguinal recurrence rate was only 2.5% for SLN-negative patients, and the 10-year disease-specific survival rate was 91% for SLN-negative patients but 65% for SLN-positive patients. The association between the size of metastatic SLNs and risk of metastases in non-SLNs was also assessed, and the rate of non-SLN metastases increased with the size of metastatic SLNs [66]. In addition, the DFS rate for patients with tumor burden > 2 mm in SLN metastases was significantly lower than that for those with tumor burden ≤ 2 mm, indicating that the size of metastatic SLNs also predicts the prognosis of vulvar SCC patients [66]. The GOG-173 study is another prospective study to evaluate the outcomes of SLN-negative patients without immediate LND, and SCC patients with tumor diameter ≥ 2 cm and ≤6 cm and with a depth of invasion > 1 mm were included [67]. In this study, the total rate of positive SLNB results was 31.6%, and the rates of positive SLNB results were 26.4% and 40.9% in patients with primary tumors 2.0–3.9 cm and 4.0–6.0 cm, respectively. The total rate of false-negative results was 8.3%, and the false-negative rate was 5.5% in patients with tumors < 4.0 cm, whereas the false-negative rate was 11.5% in patients with tumors ≥ 4.0 cm. These results suggest that SLNB may be a safe alternative to inguinal LND at least for vulvar SCC patients with tumors < 4.0 cm, although these studies are limited by the single-arm design without randomization. Because nodal recurrence is known to be fatal, accurate evaluation of SLNs is mandatory [68]. The current NCCN guidelines recommend SLNB or inguinal LND for T1b or T2 lesions without clinically enlarged lymph nodes (Table 4) [69]. According to a study by Bosquest et al., subclinical metastases were found in as many as 7% of patients with primary tumors ≤ 1.0 cm [68]. Therefore, active consideration of SLNB may be required for vulvar SCC with even small-sized tumors. In the future, prospective clinical trials focusing on small-sized tumors are desired to be performed to further elucidate this point.

##### Management after Detection of Positive SLNs in Vulvar SCC

The standard management for patients with SLN metastases was immediate LND. Whether radiotherapy is a safe alternative for LND in early-stage vulvar SCC (tumor diameter < 4 cm) was assessed in the GROINSS-V-II study [70]. The ipsilateral isolated inguinal node recurrence rate at 2 years was only 1.6% among patients with SLN micrometastases (tumor burden in SLNs ≤ 2 mm) receiving adjuvant RT of 50 Gy. This value was comparable to the isolated inguinal recurrence rate in patients with SLN micrometastases receiving LND in the GROINSS-V-I study. Among patients with SLN macrometastases (tumor burden > 2 mm), the isolated inguinal recurrence rates at 2 years were 22% and 6.9% in those receiving RT and LND, respectively. Adverse events related to RT were less severe than those related to LND. These results suggest that LND can be safely replaced by RT only in patients with SLN micrometastases, not in those with SLN macrometastases. Management of the contralateral inguinal region following unilateral SLN metastases in vulvar SCC patients is still under debate [71,72]. Ignatov et al. evaluated 62 patients who underwent contralateral LND following unilateral SLN metastases, but no contralateral non-SLN metastases were found [72]. In an analysis for the outcomes of patients with unilateral SLNB from the GROINSS-V-I and II databases, non-SLN metastases in the contralateral inguinal regions were found in only 2.9% (7/244) of the patients, which was detected through either immediate LND or recurrence after observation [71]. Although the current standard management may be contralateral LND, these studies indicate that limiting inguinal treatment to a unilateral inguinal region might be safe in cases with unilateral positive SLNB, and further studies are required to evaluate its safety [52,73].

#### 2.2.3. Penile SCC

Penile SCC also has a high potential of nodal metastases, and 20–25% of patients with clinically node-negative penile SCC harbor occult inguinal LN metastases [74]. The presence of LN metastases is one of the most important prognostic factors [75,76]. Solsona et al. reported that the rates of metastatic lymph nodes were 0% in patients with low-risk (Tis or T1G1) disease, 35% in those with intermediate-risk disease (T1G2 or G3 or T2G1), and 81% in those with high-risk disease (T2G2 or T2–3G3) (Table 5) [77]. Hughes et al. found that the rate of occult nodal metastases was 9% in T1G2 patients [78]. Previous studies reported that delayed surgical treatment of occult nodal metastases results in worse prognosis [79,80]. SLNB was also introduced in penile SCC, and a high false-negative rate of 22% per patient was reported in the initial stages of SLNB for penile SCC [81]. After some modification of methods for SLNB, Leijte showed a decrease in the false-negative rate to 4.8% per groin [82]. Lam et al. conducted a prospective study at a high-volume specialist penile cancer center and found that the false-negative rates per groin and per patient were 5% and 6%, respectively [83]. This discrepancy in the false-negative rate might have been attributable to different methods of SLNB and a distinct level of technique to detect and resect accurate SLNs among the studies, and further studies for evaluating the false-negative rate in penile SCC are needed [84]. The European Association of Urology guidelines recommend that patients with intermediate-risk and high-risk ≥ pT1G2 disease should undergo sampling of the inguinal nodes via SLNB or modified inguinal LND [85,86]. If SLN metastases are found, ipsilateral radical inguinal LND is recommended [86]. As for predictors of non-SLN metastases, de Vries et al. showed that the number of positive SLNs and the largest metastatic size in SLNs were independently associated with the positivity of non-SLNs [87]. Although it was difficult to select in which patients inguinal LND can be omitted in the previous study [87], further studies with large numbers of participants would help to select such patients.

#### 2.2.4. Anal SCC

The anal canal has a rich lymphatic system and may drain into three distinct basins: inguinal, lateral pelvic, and mesorectal LNs [88,89]. Inguinal drainage increases in regions further from the dentate line and closer to the anal verge [90,91,92,93]. Metastases to inguinal lymph nodes in anal SCC develop in 15–20% of T1/T2 diseases and in up to 50% of T3/T4 diseases, and nodal involvement is an important prognostic factor for survival (Table 6) [94,95]. According to the NCCN guidelines, local excision is recommended only in perianal SCC with T1 and well or differentiated tumors or select T2 tumors that do not involve the sphincter without clinical lymph node metastases [96]. In other cases of perianal SCC and anal canal SCC, chemoradiation therapy is a standard treatment [96]. SLNB is less established in anal SCC as compared with vulvar or penile SCC and is not recommend for either anal canal SCC or perianal SCC in the NCCN guidelines [96]. In a retrospective study, 75 of 181 patients who had uninvolved inguinal nodes at presentation received prophylactic RT to the inguinal regions [97]. The 5-year cumulative rates of inguinal recurrence were 2% and 16% in patients with prophylactic RT and those without prophylactic RT, respectively, and the current NCCN guideline indicates the inguinal regions to be included in the initial radiation field even when inguinal nodes are not clinically involved [97]. However, other studies revealed that only 7.8% of patients developed inguinal metastases among patients treated with chemoradiation that spared the inguinal lymph nodes if not macroscopically involved [98,99]. In addition, severe adverse events associated with RT to the inguinal regions occurs in about 15% of patients [98,100], and therefore, the necessity of inguinal RT is still controversial [101]. Several studies analyzed whether negative SLNB results can safely exclude the inguinal regions from the RT field, but the findings varied greatly between each study [102,103]. Therefore, further prospective studies comparing the outcomes of observation of inguinal LND with those of prophylactic inguinal RT in SLNB-negative patients are required to elucidate the role of SLNB to omit RT in anal SCC.

### 2.3. Merkel Cell Carcinoma (MCC)

#### 2.3.1. Role of SLNB and Predictive Factors for Positive SLNs in MCC

MCC is a rare and aggressive cutaneous neoplasm of neuroendocrine origin [104,105]. MCC frequently develops in the head and neck region of older adults and is known to be associated with immunosuppression and polyomavirus infection [106,107]. The status of SLN is also important for predicting prognosis in MCC, although whether SLNB obtains a survival advantage when compared with observation remains unknown [9,10]. In MCC, occult nodal metastases are detected in approximately 30% of patients who received SLNB. Some risk factors for positivity of SLNB results have been suggested [108,109]. Smith et al. reported that increasing tumor diameter and increasing tumor depth are independently predictive of positive SLNs as well as worse OS and worse DSS [109]. Immunosuppression, trunk location, tumor-infiltrating lymphocytes, and lymphovascular invasion have also been reported as predictive factors for positive SLNB results [110,111]. However, a population based on tumor characteristics in which the risk of positive SLNs is lower than the accepted cutoff value of 10% was not found in these studies, indicating that SLNB should be encouraged in all MCC patients [108,109].

#### 2.3.2. Management of MCC Patients with Negative SLNs

MCC is known to show high rates of false-negative SLNB, with rates of 14.3–19.2% [108,112]. Straker et al. revealed that patients with false-negative SLNB results were significantly associated with worse survival and that male sex, age > 75 years, and lymphovascular invasion were correlated with increased risk for false-negative SLNB results [113]. Therefore, careful nodal follow-up is required even in MCC patients with negative SLNs, especially in those who have these risk factors for false-negative SLNB results. Because of the high rate of false-negative SLNB results and the high radiosensitive characteristics of MCC, adjuvant RT to the nodal basin might be performed in patients with negative SLNs. Some studies reported low rates of regional recurrence in SLNB-negative patients who underwent adjuvant nodal RT [114,115]. In contrast, Groz et al. reported no difference in the incidence of regional recurrences in SLNB-negative patients based on the use of adjuvant radiation to the nodal basin [116]. According to the NCCN guidelines, observation of the nodal basin in MCC patients with negative SLNB results is recommended, but RT to the nodal basin may be considered in high-risk patients at increased risk for false-negative SLNB results, such as profound immunosuppression and potentially inaccurate SLN mapping [13]. Further randomized clinical studies may determine MCC patients with negative SLNB suitable for receiving RT.

#### 2.3.3. Management after Detection of Positive SLNs in MCC

Cramer et al. retrospectively analyzed the outcomes of patients with positive SLNs using the National Cancer Database and reported that observation or CLND alone was associated with worse OS than that of CLND + RT after adjusting for the clinicopathologic difference, whereas adjuvant RT alone showed comparable OS to that of CLND + RT [117]. In another retrospective study, multivariate analysis revealed that SLN status and RT for the primary tumor with or without regional lymph nodes were significantly associated with better DFS and OS [118]. Sims et al. also demonstrated that patients with a positive SLN have a higher risk of in-transit recurrence, suggesting that such patients may benefit from adjuvant RT with inclusion of the in-transit field [119]. The current NCCN guidelines recommend LND and/or RT for the treatment of patients with positive SLNs [13]. However, on the basis of these clinical studies, RT should be actively considered not only for primary lesions but also for regional node basins regardless of whether LND is performed, and inclusion of the in-transit regions also should be considered when the primary lesions are close to their nodal basin. In this regard, further randomized prospective studies for evaluating the effect of adjuvant RT after detection of positive SLNs might determine the impact of adjuvant RT as compared with that of LND in the prognosis of MC patients in the future.

### 2.4. Extramammary Paget Disease (EMPD)

EMPD is a rare neoplasm that usually develops in the apocrine gland-bearing areas of older people [120,121]. Most EMPD cases are diagnosed as carcinoma in situ, which usually shows indolent disease progression. However, once Paget cells invade deeply into the dermis, regional lymph node metastases and distant metastases frequently develop. Individuals with distant metastases have a poor prognosis because of the limited efficacy of chemotherapies [122]. The role of SLNB in EMPD has not been well established, and most previous reports regarding SLNB in EMPD were case reports or retrospective studies with relatively small patient numbers [123,124,125]. Fujisawa et al. conducted a multicenter retrospective study of 151 invasive EMPD patients who underwent SLNB [122]. They found that the overall rate of positive SLNB results in patients with invasive EMPD without lymphadenopathy was 15% and that the independent factors associated with this positive rate were invasion into the reticular dermis or deeper and presence of lymphovascular invasion. Although SLN status did not have an impact on survival, this might have been attributable to improved survival as a result of LND in SLNB-positive patients. Maeda et al. also evaluated the outcomes of invasive EMPD patients who underwent SLNB [126]. In their study, sentinel lymph node metastases were classified as macrometastases and micrometastases, with a cutoff value for SLN of 2 mm, and this status of SLNB was an independent prognostic factor for RFS. They also demonstrated that invasion level (invasion within the papillary dermis vs. invasion into the reticular dermis or deeper) and a high neutrophil-to-lymphocyte ratio were independent predictive factors for positivity of SLNB results. However, a population based on tumor characteristics in which the risk of positive SLNs is lower than the accepted cutoff value of 10% was not found in these studies. In contrast, the false-negative rate in EMPD has been reported to be low, at 6–8% [124,126]. Therefore, given that SLNB is a relatively safe procedure [121,122], simultaneous SLNB with primary tumor resection might be worth considering in EMPD cases with clinical findings suspicious for invasion including microinvasion, such as small ulceration and slightly elevated plaques. However, further studies, especially prospective studies with high quality, are desired to be conducted to clarify the role of SLNB in EMPD since available evidence is currently limited.

### 2.5. Sebaceous Carcinoma (SC)

SC is a rare and aggressive tumor with potential for LN and distant metastases [127,128]. It frequently develops on the eyelid, called periocular SC, but SC may also occur outside the eyelid, called extraocular SC [128]. Extraocular SC develops mainly on the head and neck [127]. Guidelines suggest that AJCC-8 stage T2c or greater tumors of periocular SC (tumors involving the full thickness of the eyelid or with more than 20 mm in greatest dimension) can be considered for SLNB, but routine use of SLNB in extraocular SC is not recommended [129]. Tryggvason et al. reported that the overall rate of nodal metastases in SC was only 2.4%, but this did not include occult LN metastases detected through SLNB [130]. Maloney also conducted retrospective studies including both periocular and extraocular SC using the National Cancer Database [128]. In this study, multivariate analyses revealed that high histologic grade, tumor size > 2 cm, Medicaid/no insurance, and periocular primary site versus the trunk/extremities were independent predictors of advanced disease [128]. Among patients with localized disease who underwent SLNB, the overall rate of positive SLNB results was 7.4% and the rate of false-negative results was 26.7% [128]. The rate of positive SLNB results in extraocular SC was only slightly lower than that in periocular SC, and approximately two-thirds of the patients with positive SLNs had a high histologic grade [128]. Therefore, SLNB can be considered for patients with a large tumor or high histologic grade of extraocular SC. However, further studies should be conducted to determine the role of SLNB in both periocular and extraocular SC.

### 2.6. Basal Cell Carcinoma (BCC)

Although BCC is the most common skin cancer with a potential risk for local invasion and recurrence, metastases including lymph node metastases are quite rare [131,132,133,134]. The annual incidence of metastasis is estimated to range between 0.0028 and 0.05% [131,133,134]. Therefore, SLNB is not usually applied for BCC. However, a few cases in which occult lymph node metastases were detected via SLNB in BCC with high-risk features including lymphovascular invasion have also been reported [131,135]. Further studies are required for evaluating the potential use of SLNB in BCC.

### 2.7. Other Skin Cancers

SLNB may be useful in other skin cancers with a high nodal metastatic potential. However, only case reports or retrospective studies with small numbers of patients who received SLNB have been published. Tsunoda et al. reported that three of eight patients with eccrine porocarcinoma who underwent SLNB were positive for metastases [136]. In contrast, Storino et al. reviewed the outcome of SLNB in malignant adnexal tumors and found that only 1 of 25 patients who underwent SLNB was positive for SLN metastasis [137]. These included apocrine carcinoma (1 of 3), digital papillary adenocarcinoma (0 of 8), porocarcinoma (0 of 4), and other adnexal tumors (0 of 10) [137]. In the future, studies with large numbers of patients and with evaluation of false-negative rates are required to determine the role of SLNB in these skin cancers.

## 3. Conclusions

In this review, we have summarized the evidence from previous studies related to the role of SLNB in skin cancer. The indication of use of SLNB for each cancer is summarized in Table 7. Use of SLNB was first established for melanoma, and the MSLT-I trial demonstrated a prognosis benefit conferred by SLNB [11,12]. SLNB has also been spread to other skin cancers as a minimally invasive and relatively safe staging technique. However, the role of SLNB in nonmelanoma skin cancers was mainly evaluated through retrospective studies. Although single-arm prospective studies for evaluating the role of SLNB in some nonmelanoma skin cancers have also been conducted, no randomized clinical trials have been reported [63,64,65,138]. Therefore, the benefit of SLNB for prognosis in patients with nonmelanoma is largely unclear although SLNB provides important information for nodal staging. In addition, the treatment choice for SLNB-positive patients is still a matter of debate. Furthermore, novel drugs have been rapidly emerging for treatment of not only melanoma but also nonmelanoma skin cancers [139,140]. Therefore, further, high-quality studies including randomized clinical trials in accordance with these novel drugs are necessary to determine the significance of SLNB for prognosis and appropriate methods or treatment according to the SLN status in skin cancer.

We hope that this review provides an up-to-date overview of SLNB in skin cancer and that it facilitates the determination of SLNB in clinical practice. Some tumors introduced in this review such as melanoma, SCC, and EMPD may be treated by not only dermatologists but also doctors of other departments including gynecologists, urologists, gastroenterological surgeons, and otolaryngological surgeons. Although the concept of the role of SLNB in each cancer may differ significantly among the doctors of different departments, we nevertheless hope that this review fills in the gap.

## Figures and Tables

**Table 1:** Recent studies evaluating predictive factors for positive sentinel lymph nodes in thin melanoma.

Year	Researchers	Study Design	Inclusion Criteria	Number of Patients	Predictive Factors for Positive Sentinel Lymph Nodes (Positive Rate)
2022	Walker et al. [41]	Retrospective multi-institutional study	Thickness ≤ 1.0 mm	676	Thickness <0.75 (2.5%) 0.75–0.84 (6.3%) 0.85–0.94 (9.8%) >0.95 (10.3%) Mitotic index/mm^2^ (9.1%) 0–1 (4.5%) 2–3 (9.1%) >3 (17.3%)
2022	Huang et al. [42]	Meta-analysis	Thickness ≤ 1.0 mm	38,844	Thickness ≥ 0.8 mm (7.0%) Ulceration (4.2%) Mitotic index > 0/mm^2^ (7.7%)
2023	Shannon et al. [43]	Retrospective multi-institutional study	Thickness < 0.8 mm, nonulcerated	965	Age ≤ 42 years (7.5%) Head/neck primary tumor location (9.2%) Lymphovascular invasion (21.4%) Mitotic index ≥ 2/mm^2^ (8.2%)

**Table 2:** Primary tumor stage of cutaneous squamous cell carcinoma according to the eighth edition of the American Joint Committee on Cancer Manual.

Primay Tumor	Criteria
T1	<2 cm in greatest diameter
T2	≥2 cm but <4 cm in greatest diameter
T3	Tumor ≥ 4 cm in greatest diameter or minor bone invasion or perineural invasion or deep invasion *
T4a	Tumor with gross cortical bone and/or marrow invasion
T4b	Tumor with skull bone invasion and/or skull base foramen involvement

* Deep invasion defined as invasion beyond the subcutaneous fat or >6 mm (as measured from the granular layer of the adjacent normal epidermis to the base of the tumor), perineural invasion defined as tumor cells in the nerve sheath of a nerve lying deeper than the dermis or measuring ≥ 0.1 mm in caliber, or presenting with clinical or radiographic involvement of named nerves without skull base invasion or transgression.

**Table 3:** Primary tumor staging of cutaneous squamous cell carcinoma according to the Brigham and Women’s Hospital system.

Primary Tumor	Criteria *
T0	In situ SCC
T1	0 risk factor
T2a	1 risk factor
T2b	2–3 risk factors
T3	4 risk factors or bone invasion

* Risk factors include tumor diameter ≥ 2 cm, poorly differentiated histologic characteristics, perineural invasion, and tumor invasion beyond the subcutaneous fat (excluding bone invasion, which automatically upgrades the tumor to alternative stage T3).

**Table 4:** Primary tumor stage of vulvar cancer according to the eighth edition of the American Joint Committee on Cancer Manual.

Criteria	Criteria
Tis	Carcinoma in situ (preinvasive carcinoma) *
T1a	Lesions ≤ 2 cm in size, confined to the vulva or perineum and with stromal invasion ≤ 1.0 mm **
T1b	Lesions > 2 cm in size or any size with stromal invasion > 1.0 mm, confined to the vulva or perineum
T2	Tumor of any size with extension to the adjacent perineal structures (lower/distal 1/3 urethra, lower/distal 1/3 vagina, anal involvement)
T3	Tumor of any size with extension to any of the following: upper/proximal 2/3 of urethra, upper/proximal 2/3 vagina, bladder mucosa, rectal mucosa, or fixed to pelvic bone

The definitions of the T categories correspond to the stages accepted by the Fédération Internationale de Gynécologie et d’Obstétrique (FIGO). * The FIGO no longer includes Stage 0 (Tis). ** The depth of invasion is defined as the measurement of the tumor from the epithelial–stromal junction of the adjacent most superficial dermal papilla to the deepest point of invasion. The FIGO uses the classification T2/T3, which is defined as T2 in the TNM classification. The FIGO uses the classification T4, which is defined as T3 in the TNM classification.

**Table 5:** Primary tumor stage of penile cancer according to the eighth edition of the American Joint Committee on Cancer Manual.

Primary Tumor	Criteria
Tis	Carcinoma in situ
Ta	Noninvasive localized squamous cell carcinoma
T1	Extent of tumor invasion location dependent: Glans: Tumor invades lamina propria Foreskin: Tumor invades dermis, lamina propria, or dartos fascia Shaft: Tumor invades connective tissue between epidermis and corpora regardless of location
T1a	Tumor is without lymphovascular invasion or perineural invasion and is not high grade (T1G1–2)
T1b	Tumor exhibits lymphovascular invasion and/or perineural invasion or is high grade (T1G3–4)
T2	Tumor invades corpus spongiosum (glans or ventral shaft) with or without urethral invasion
T3	Tumor invades corpora cavernosum (including tunica albuginea) with or without urethral invasion
T4	Tumor invades into adjacent structures (e.g., scrotum, bladder wall)
Histologic grade	Criteria
GX	Grade of differentiation cannot be assesed
G1	Well differentiated
G2	Moderately differentiated
G3–4	Poorly differentiated/undifferentitated

**Table 6:** Primary tumor stage of anal cancer according to the eighth edition of the American Joint Committee on Cancer Manual.

Primary Tumor	Criteria
Tis	Carcinoma in situ (Bowen disease, high-grade squamous intraepithelial lesion [HSIL], anal intraepithelial neoplasia II–III [AIN II–III])
T1	≤2 cm in greatest diameter
T2	>2 cm, but ≤5 cm in greatest diameter
T3	Tumor > 5 cm in greatest diameter
T4	Tumor of any size invades adjacent organ(s), e.g., vagina, urethra, bladder *

* Direct invasion of the rectal wall, perirectal skin, subcutaneous tissue, or sphincter muscle(s) is not classified as T4.

**Table 7:** Indication of SLNB in each tumor.

Tumor Type	Indication (Guidelines or Our Suggestion Based on Previous Studies)
Melanoma	The current NCCN guidelines recommend SLNB to be offered in melanoma patientsa with T2a or higher lesions and to be considered in melanoma patients with T1b lesions and T1a lesions with other adverse features such as mitotic index ≥ 2/mm^2^, particularly in the setting of young age and lymphovascular invasion [40].
Cutaneous SCC	The current NCCN guidelines suggest SLNB to be considered in very-high-risk cutaneous SCCs that are recurrent or have mutiple risk factors for local recurrence, metastases, or death from disease [14]. We suggest SLNB to be considered for at least T2b lesions according to the BWH staging system.
Vulvar SCC	The current NCCN guidelines recommend SLNB or inguinal LND for T1b or T2 lesions without clinically enlarged lymph nodes [69].
Penil SCC	The European Association of Urology guidelines recommend that patients with intermediate-risk and high-risk ≥ pT1G2 disease should undergo sampling of the inguinal nodes via SLNB or modified inguinal LND [85,86].
Anal SCC	The current NCCN guidelines do not recommend SLNB for either anal canal SCC or perianal SCC [96].
MCC	The current NCCN guidelines recommend SLNB for MC patients without clinical findings of metastases [13].
EMPD	We suggest that SLNB might be worth considering in EMPD cases with clinical findings suspicious for invasion.
SC	Evidence-based clinical practice guidelines suggest that T2c or greater tumors of periocular SC can be considered for SLNB [129]. We suggest that SLNB can be considered for patients with a large tumor or high histologic grade of extraocular SC.

SCC: squamous cell carcinoma, MCC: Merkel cell carcinoma, EPMD: extramammary Paget disease, SC: sebaceous carcinoma, NCCN: National Comprehensive Care Network, SLNB: sentinel lymph node biopsy, LND: lymph node dissection, BWH: Brigham and Women’s Hospital.
